# Mercuric Chloride

**Published:** 1890-02

**Authors:** 


DR. SCALGI, of Rome, recommends for antisepsis, very weak solutions of mercuric chloride at a temperature of 40 or 50 degrees C. as being more active than ordinary solutions now used in surgery.

These, according to the *Annales d'Orthopedie*, produce at the surface of the wound, a coagulation with union by first intention. The strength employed is one to 10,000 or 20,000.

